# tropiTree: An NGS-Based EST-SSR Resource for 24 Tropical Tree Species

**DOI:** 10.1371/journal.pone.0102502

**Published:** 2014-07-15

**Authors:** Joanne R. Russell, Peter E. Hedley, Linda Cardle, Siobhan Dancey, Jenny Morris, Allan Booth, David Odee, Lucy Mwaura, William Omondi, Peter Angaine, Joseph Machua, Alice Muchugi, Iain Milne, Roeland Kindt, Ramni Jamnadass, Ian K. Dawson

**Affiliations:** 1 Cell and Molecular Sciences, James Hutton Institute, Invergowrie, Scotland, United Kingdom; 2 Information and Computational Sciences, James Hutton Institute, Invergowrie, Scotland, United Kingdom; 3 College of Life Sciences, University of Dundee, Dundee, Scotland, United Kingdom; 4 Headquarters, Kenya Forestry Research Institute, Nairobi, Kenya; 5 Centre for Ecology & Hydrology at Edinburgh, Centre for Ecology & Hydrology, Penicuik, Scotland, United Kingdom; 6 Headquarters, World Agroforestry Centre, Nairobi, Kenya; National Institute of Plant Genome Research, India

## Abstract

The development of genetic tools for non-model organisms has been hampered by cost, but advances in next-generation sequencing (NGS) have created new opportunities. In ecological research, this raises the prospect for developing molecular markers to simultaneously study important genetic processes such as gene flow in multiple non-model plant species within complex natural and anthropogenic landscapes. Here, we report the use of bar-coded multiplexed paired-end Illumina NGS for the *de novo* development of expressed sequence tag-derived simple sequence repeat (EST-SSR) markers at low cost for a range of 24 tree species. Each chosen tree species is important in complex tropical agroforestry systems where little is currently known about many genetic processes. An average of more than 5,000 EST-SSRs was identified for each of the 24 sequenced species, whereas prior to analysis 20 of the species had fewer than 100 nucleotide sequence citations. To make results available to potential users in a suitable format, we have developed an open-access, interactive online database, tropiTree (http://bioinf.hutton.ac.uk/tropiTree), which has a range of visualisation and search facilities, and which is a model for the efficient presentation and application of NGS data.

## Introduction

In the last decades, agroforestry practices that integrate trees in agricultural landscapes have received increased attention for their ecosystem functions including biodiversity conservation [1]. This is especially so in the context of expanding global challenges to food production and the environment such as climate change, soil fertility depletion and forest loss [2]. From a biodiversity-maintenance perspective, the persistence of trees in farm landscapes depends on their regenerational behaviour, which is influenced by levels of genetic diversity and by gene flow [3]. Most tree species, for example, are predominantly outbreeding and can suffer from inbreeding depression if landscape genetic diversity and connectivity are not maintained [4].

The relatively limited evidence assembled so far suggests that some tree species in farmland have passed through significant genetic diversity bottlenecks, while others have not, depending in part on the primary function allocated to each species by farmers and the source of planting material (reviewed in [5]). In addition, while gene flow may be higher among farmland trees than in natural landscapes, it can also be reduced, depending in part on tree density [5]. A crucial aspect of many tropical farms is their very high tree species diversity [6]. Positive and negative interactions occur between the various species in these systems [7], however, and further exploration of the importance of these landscapes for conservation therefore requires parallel genetic-level research on a wide range of tree species within them.

Until recently, parallel research on multiple tree species within systems has been hampered by the slow rate of development of appropriate tools for genetic assessment, reflecting the prohibitive costs involved. With the rapid development of next-generation sequencing (NGS) technologies, however, the ability to develop molecular markers for non-model organisms has been enormously enhanced [8]–[11]. The proper application of NGS data still, however, requires that appropriate ways to visualise and manipulate data are described.

In this study, our objectives were two-fold. First, we wished to rectify the absence of genetic tools for a range of important tree species that are often found co-occurring in key tropical agroforestry landscapes. Second, we wished to present the NGS data so generated in a format suitable for efficient use by scientists who are not necessarily familiar with modern sequence-based molecular technologies. To these ends, we first used bar-coded multiplexed paired-end Illumina next-generation sequencing of RNA to develop expressed sequence tag-derived simple sequence repeat (EST-SSR) markers for a range of 24 tree species of importance to tropical smallholders. We then presented results in a specially developed, inter-relational open-access online RNA-Seq database that we have called tropiTree (http://bioinf.hutton.ac.uk/tropiTree).

The low-cost sequencing method applied here resulted on average in more than 5,000 EST-SSRs being identified for each of the sequenced tree species, with a mean of more than 4,000 putative primer pairs designed to EST-SSRs in each case. This represents a resource far greater than that required for most standard population genetic applications, providing the potential to study genetic variation in subsamples of selected sequences. Complete sequence data and assemblies can be downloaded from tropiTree into Tablet, a lightweight high-performance graphical viewer designed by the James Hutton Institute (JHI) for NGS alignments and for further manipulations [12].

## Materials and Methods

### Choice of Species

Twenty-four trees of value to tropical smallholders were chosen from a much larger range of species listed by the World Agroforestry Centre’s (ICRAF’s) Agroforestree Database (Table 1), based on three main criteria: 1) species were identified as priorities for research through discussions with ICRAF’s research scientists and national partners in Africa, Asia and Latin America; 2) seed of species were of orthodox (or at worst intermediate) storage behaviour, so that they could be transported to JHI in the UK for RNA extraction without loss of germination capacity; and 3) seed had to be available for shipment to JHI from the wide-ranging tree germplasm collections held by ICRAF and the Kenya Forest Seed Centre (at the Kenya Forestry Research Institute, KEFRI) in Kenya.

**Table 1:** Information on 24 tropical trees subjected to next-generation sequencing and screened for SSRs.

Species	Primary use^a^	Origin^a^	Geographic source ofmaterial for NGS^b^	Transcriptsassembled^c^	Total Mbp^c^	SSRsidentified^c^	Putative primerpairs^c^	NCBI nucleotide sequence citations^d^
*Acacia mangium* e	Timber	Australia, SE Asia	Nalgonda, India, 04713	56,655	42.3	8,294 (3,400) [64]	6,778	9,282 (9,110) [2]
*Acacia senegal*	Gum (gum arabic)	Sub-Sahar. Africa	Cherangani, Kenya, 04991	36,996	22.8	4,151 (1,650) [70]	3,407	72
*Acrocarpus fraxinifolius*	Timber, shade	Asia	Muringato, Kenya, 05053	54,918	38.0	6,566 (2,361) [68]	5,200	14
*Adansonia digitata*	Fruit, vegetable	Sub-Sahar. Africa	Kibwezi, Kenya, 02910	6,873	2.9	461 (156) [59]	372	46
*Albizia lebbeck*	Timber, fuelwood	Australia, Asia	Gambari, Nigeria, n/a	53,311	37.3	7,764 (3,369) [64]	6,110	23
*Calliandra calothyrsus*	Fodder	Mex., Cent. Amer.	Muguga, Kenya, 04873	46,619	33.6	7,851 (3,441) [68]	6,341	11
*Diospyros mespiliformis*	Fruit	Africa	Kibwezi, Kenya, 05655	36,701	23.3	6,980 (3,646) [67]	4,322	19
*Enterolobium cyclocarpum*	Timber, fodder	Mex., Cent. andS. Amer.	West Java, Indonesia, 05055	50,511	41.2	7,079 (3,018) [68]	5,681	32
*Faidherbia albida* f	Fodder, soil fertility	Middle East, Africa	Taveta, Kenya, n/a	25,253	10.5	1,711 (655) [58]	1,404	27
*Gliricidia sepium*	Fodder, soil fertility	Mex., Cent. Amer.	Morogoro, Tanzania, 04891	44,622	28.5	7,421 (3,163) [71]	5,607	25
*Jacaranda mimosifolia*	Shade, ornamental	S. America	Sirisia, Kenya, 05669	51,525	36.2	6,282 (2,479) [66]	4,727	21
*Jatropha curcas* e	Biodiesel	Mex., Cent. Amer.	Shimba Hills, Kenya, 04845	13,252	5.3	1,118 (359) [57]	863	120,096 (46,865) [10]
*Leucaena diversifolia*	Fuelwood, shade	Mex., Cent. Amer.	Machakos, Kenya, 03356	55,714	30.5	6,193 (2,454) [59]	5,021	80
*Leucaena leucocephala*	Fodder	Mex., Cent. Amer.	Machakos, Kenya, 05672	46,231	26.6	5,285 (2,170) [68]	4,396	484 (150)
*Moringa stenopetala*	Vegetable	E. Africa	Kitale, Kenya, 04877	31,408	23.6	5,239 (1,957) [79]	3,954	3
*Prunus africana* f	Medicine	Sub-Sahar. Africa	Kaplamai, Kenya, 05670	1,976	1.2	117 (32) [67]	84	201
*Samanea saman*	Timber, shade	Cent. and S. Amer.	West Java, Indonesia, 05056	38,843	25.5	5,102 (2,135) [71]	4,070	47
*Senna siamea*	Fuelwood, shade	Asia	Kisumu, Kenya, 03115	54,207	40.8	9,067 (3,697) [70]	7,177	12
*Sesbania macrantha*	Fodder, soil fertility	E. and southern Africa	Baba-Kuru, Nigeria, 00151	32,618	19.0	4,183 (1,512) [70]	3,476	3
*Sesbania sesban*	Fodder, soil fertility	Africa	Muguga, Kenya, 03276	33,306	19.5	4,308 (1,639) [71]	3,565	14
*Tephrosia candida*	Soil fertility	India	Maseno, Kenya, 03116	33,586	22.0	6,509 (2,723) [74]	4,809	6
*Tipuana tipu*	Shade, ornamental	S. Amer.	Muguga, Kenya, 01897	33,331	19.7	6,057 (2,621) [67]	4,550	12
*Warburgia ugandensis*	Medicine	E. Africa	Mt. Elgon For., Kenya, 05671	19,241	9.2	1,796 (766) [65]	1,330	16
*Ziziphus mauritiana*	Fruit	Africa, Asia	West Pokot, Kenya, 04626	27,968	16.9	5,201 (2,271) [75]	3,518	88

a Based on the Agroforestree Database (www.worldagroforestry.org/resources/databases/agroforestree), an open access resource of ICRAF that provides data on >650 trees.

b The seed source of material for NGS varied and included natural stands, seed orchards and landraces. The numerical reference is the ICRAF accession number.

c Current data from NGS; complete information is available at the tropiTree portal (http://bioinf.hutton.ac.uk/tropiTree). In () is the number of perfect SSRs identified. In [] is the percentage of the corresponding transcripts that have TAIR hits (for all SSRs).

d Data from National Center for Biotechnology Information of the USA (NCBI) searches were included to illustrate previous sequencing work. Searches were undertaken on 14 April 2014 via the Entrez search system (www.ncbi.nlm.nih.gov/sites/gquery). Species names for NCBI searches were checked as correct against current nomenclature using the Agroforestry Species Switchboard (www.worldagroforestry.org/products/switchboard/), an open access resource of ICRAF that provides links to information on >20,000 plants. Current names were set as ‘organism’ in NCBI searches. In () is the number of ESTs listed in NCBI nucleotide citations (if any). In [] is the number of NGS studies cited in NCBI’s Sequence Read Archive (if any).

e As well as being of importance to small-scale farmers, *Acacia mangium* and *Jatropha curcas* have wide commercial interests (see text), explaining the high NCBI citations.

f Species were subject to primer validation (see text).

The final list of chosen tree species included nine of solely African origin, five from Asia/Oceania, one with a natural distribution spanning both Africa and Asia, and nine from Latin America (Table 1). Due to human movement of germplasm, the selected species are now often found growing together in various combinations of indigenous and exotic trees in agricultural landscapes. As outlined in Table 1, they fulfil a range of primary functions for farmers, such as animal fodder, fruit for human consumption, medicines, soil fertility replenishment and timber. The densities and configurations of these tree species in farmland vary, depending on the particular uses assigned to them by farmers, their biologies and the type of agroforestry system of which they are part; they also exist in different relationships to natural forests that may or may not contain the same trees [5].

Most of the chosen tree species are only incipient domesticates, although a few such as *Acacia mangium*, *Jatropha curcas*, *Leucaena leucocephala* and *Ziziphus mauritiana* have been subject to a degree of formal breeding. Even for these species, however, many of the trees found planted in smallholders’ fields are ‘landraces’ of unknown provenance, due to the highly informal nature of germplasm sourcing in the tropical agroforestry sector [13]. Their genetic constitution and behaviour on farm are therefore little known.

### RNA Extraction and Sequencing

All legal and phytosanitary requirements for the export and import of seed were followed in transport to JHI. Seed were germinated on moist filter paper or 1% agarose after applying specialised pretreatments to enhance germination, where required (depending on seed size and biology; see www.worldagroforestry.org/resources/databases/agroforestree). Following germination, dissected embryonic tissue (further information in Table S1) was flash frozen in liquid nitrogen. Germinated seed were used for RNA extraction because our previous experience demonstrated that they provide a wide range of transcripts [36]. For each species, total RNA was extracted from 200 mg of ground frozen tissue, using 2 ml TriReagent (Sigma-Aldrich) as recommended by the manufacturer, with additional phenol-chloroform purification steps and ethanol precipitation.

Extracted RNAs were quality checked using the RNA 6000 Nano kit on a 2100 Bioanalyzer (Agilent). One µg samples of RNA of each species (except in the case of *Prunus africana*, for which 200 ng was used due to poor RNA recovery during extraction) were submitted to Glasgow Polyomics, University of Glasgow, for the generation of RNA-Seq data. TruSeq RNA (Illumina) libraries were made using manufacturer-recommended protocols and indexed to allow 12 libraries to be combined in a single lane (i.e., 12 tree species per lane) of an Illumina GAII run. Paired-end 110 or 73 bp reads (runs FC088 and FC095, respectively) were obtained from two lanes in total for the 24 species.

### Sequence Assembly and Analysis

Raw FASTQ files were quality trimmed using the ‘quality_trim’ utility from the CLC bio Assembly Cell (CLC Assembly Cell 4.0 [14]) to a minimum length of 25 bp and a minimum quality score (Phred) of 20, as specified in the user manual [15]. Each sample was *de novo* assembled with Trinity (version trinityrnaseq_r2012-06-08 [16]) with default settings. SSRs were detected in Trinity consensus sequences using Phobos (version 3.3.10 [17]) using the ‘–M extendExact’ option to search for di-, tri- and tetra-nucleotide repeats equal to or greater than 12, 15 and 16 bp in length, respectively (i.e., ≥6, 5 and 4 repeats of the motif, respectively). Other nucleotide repeat motifs were not considered during detection.

Primer3 (version 1.1.1 [18]) was used to design primers around each located SSR based on default settings except for the following: ‘PRIMER_OPT_TM’ = 55.0; ‘PRIMER_MIN_TM’ = 50.0; ‘PRIMER_MAX_TM’ = 60.0; ‘PRIMER_MIN_GC’ = 30; ‘PRIMER_MAX_GC’ = 70; and ‘PRIMER_PRODUCT_SIZE_RANGE’ = 150–250. Consensus sequences were annotated by a BLASTX search (version 2.2.26 [19]) against TAIR v10 pseudo-peptides (www.arabidopsis.org/) with a minimum e-value cut off of 1e-10. An inter-relational online database was specially designed to present results (http://bioinf.hutton.ac.uk/tropiTree).

### Marker Validation

For two of the 24 species, *Faidherbia albida* and *P*. *africana*, we tested the utility of EST-SSRs as markers against panels of individuals taken from the tree population used for NGS, supplemented by seedlings from another proximate population (in order to enhance the prospects for discovering polymorphism). Both of the species chosen for validation are of African origin, are diploid, and are the subject of current active research because of the import products and services they provide to local communities in sub-Saharan Africa [20]–[23]. DNA for testing was extracted from dried or fresh leaf material of individual seedlings using the Qiagen DNeasy kit.

For testing, subsets of primer pairs for SSRs were chosen based on the following criteria: 1) repeat of the motif was at least seven and six times for di- and tri-nucleotides, respectively (the original criterion of 4 repeats for the tetra-nucleotide motif was retained); and 2) repeat perfection in at least 90% of the sequence. In the case of *F*. *albida*, many sequences met these criteria, so primer pairs were then sampled at random for testing. These criteria were however sometimes relaxed for *P. africana* because of the small total number of SSRs identified in this instance (see more below).

Initially, 44 primer sets for *F. albida* and 40 for *P. africana* were tested on a panel of eight individuals of each species (Table S2). Loci were amplified individually in 10 µl reactions containing 50 ng of DNA template with a Gene Amp PCR System 9700 thermo cycler (Applied Biosystems), using Hot Start Taq (Roche Applied Science) and standard protocols [24]. The following PCR profile was used: 95°C for 15 min; 94°C for 30 s, 54–58°C for 35–45 s, 72°C for 30 s, 35 cycles; 72°C for 5 min. PCR products were initially run on 1.5% agarose gels. For promising primers (those that revealed clear product of approximately the expected size across the initial test panel, see Table S2), the forward primer was fluorescently labelled, PCR undertaken on a larger panel of 30 individuals, and products separated and sized with an ABI 3730 DNA analyser and GeneMapper software, based on standard protocols (Applied Biosystems).

## Results

All sequence and primer data reported below are available through the tropiTree online portal (http://bioinf.hutton.ac.uk/tropiTree). In addition, sequence data are available at the European Nucleotide Archive under the following accession numbers: PRJEB5301 (study accession number, see www.ebi.ac.uk/ena/data/view/PRJEB5301); ERS399684 (*Acacia mangium*, this and the following references = the sample accession number for the given species); ERS399685 (*Acacia senegal*); ERS399686 (*Acrocarpus fraxinifolius*); ERS399687 (*Adansonia digitata*); ERS399688 (*Albizia lebbeck*); ERS399689 (*Calliandra calothyrsus*); ERS399690 (*Diospyros mespiliformis*); ERS399691 (*Enterolobium cyclocarpum*); ERS399692 (*Faidherbia albida*); ERS399693 (*Gliricidia sepium*); ERS399694 (*Jacaranda mimosifolia*); ERS399695 (*Jatropha curcas*); ERS399696 (*Leucaena diversifolia*); ERS399697 (*Leucaena leucocephala*); ERS399698 (*Moringa stenopetala*); ERS399699 (*Prunus africana*); ERS399700 (*Samanea saman*); ERS399701 (*Senna siamea*); ERS399702 (*Sesbania macrantha*); ERS399703 (*Sesbania sesban*); ERS399704 (*Tephrosia candida*); ERS399705 (*Tipuana tipu*); ERS399706 (*Warburgia ugandensis*); and ERS399707 (*Ziziphus mauritiana*).

For each of the 24 tree species, the number of transcripts assembled following sequencing, the total Mbp sequenced, the number of SSRs identified (di-, tri- and tetra-nucleotide repeats combined) and the number of putative primer pairs for SSRs are summarised in Table 1. Across the 24 species, averages of 36,903 transcripts and 24 Mbp of sequence were assembled, ranging from 1,976 transcripts and 1.2 Mbp of sequence for *P. africana* (for which only 20% of the amount of RNA was sequenced compared to other species, see above) to 56,655 transcripts and 42.3 Mbp of sequence for *A. mangium*.

Across species, a mean of 5,197 SSRs was identified, ranging from 117 SSRs for *P. africana* to 9,067 SSRs for *Senna siamea*. Over all species, SSRs were observed on average once every 4,987 bp of sequence, ranging from one SSR every 3,249 bp for *Z*. *mauritiana* to one every 10,256 bp for *P*. *africana* (low occurrence in the latter case could be a reflection of poor sequence assembly over a limited number of reads). Two pairs of related species showed very similar frequencies for SSR occurrence, *Leucaena diversifolia* and *L*. *leucocephala* (every 4925 and 5033 bp, respectively), and *Sesbania macrantha* and *Sesbania sesban* (every 4542 and 4526 bp, respectively). Across species, an average of 2,153 SSRs (40%) represented perfect repeats (i.e., a particular motif repeated in an uninterrupted array), with the lowest proportion of perfect SSRs for *P. africana* (27%) and the highest for *Diospyros mespiliformis* (52%). On average, 67% of the corresponding transcripts to SSRs had TAIR hits, ranging from 57% (*J. curcas*) to 79% (*Moringa stenopetala*).

**Figure 1:**
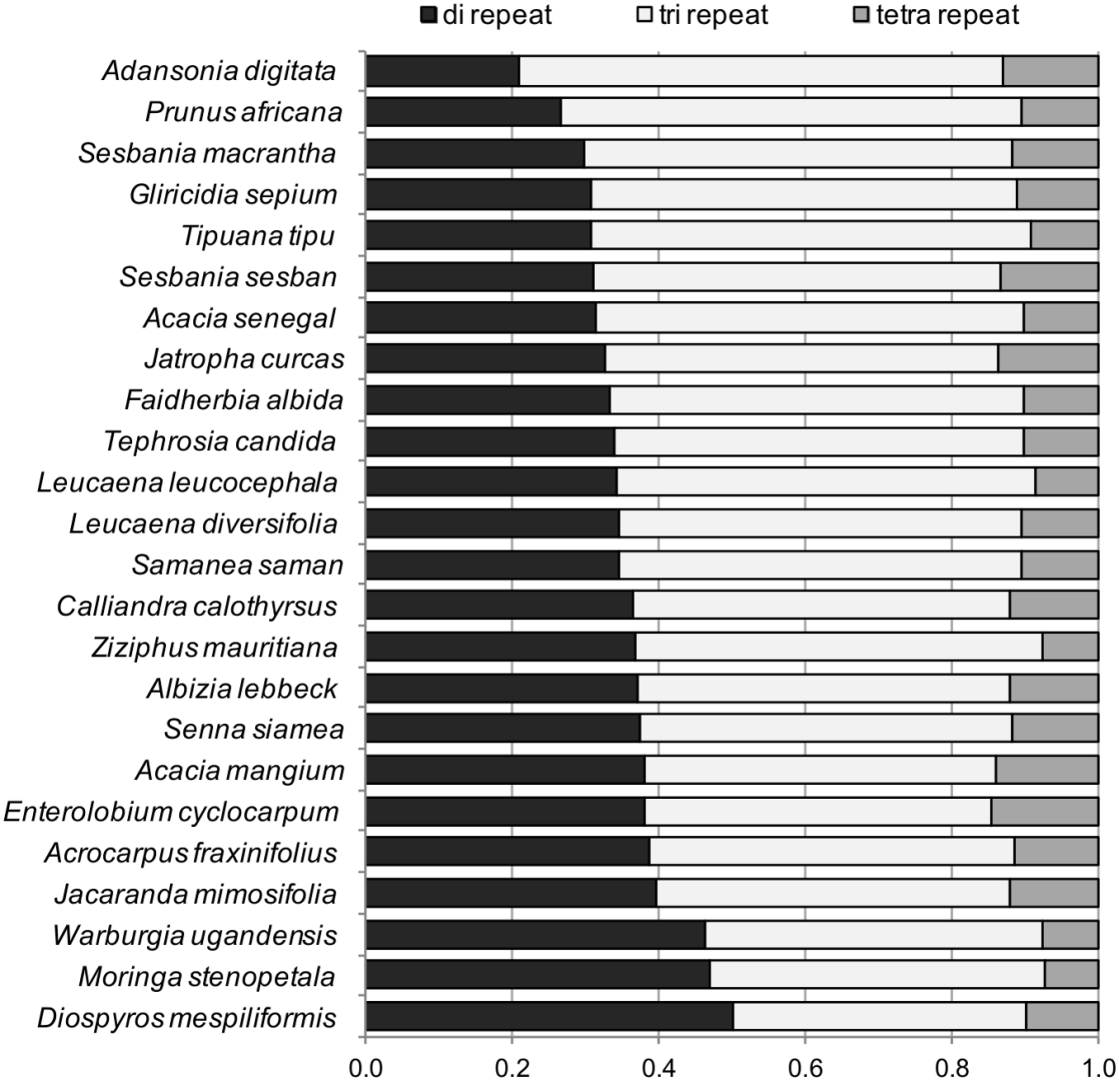
SSR repeats in 24 tree species subjected to next-generation sequencing. The proportion of di-, tri- and tetra-nucleotide repeats is shown. The species are ordered by the proportion of di-nucleotide repeats revealed.

Using Primer3, a mean across species of 4,032 putative primer pairs was designed to EST-SSRs. As expected, linear regression analysis of: 1) the number of transcripts assembled; 2) the total Mbp sequenced; 3) the number of perfect SSRs identified; and 4) the number of putative primer pairs to SSRs, all versus the total number of SSRs identified, showed highly significant positive correlations (*R^2^* = 0.81, 0.84, 0.97 and 0.98, respectively, *P*<0.0001 in each case). On average, 35% of the total identified SSRs were di-, 54% tri- and 11% tetra-nucleotide repeats, with some variation in the proportion of each type of repeat observed across species (Fig. 1). *Adansonia digitata* and *D*. *mespiliformis* had the lowest and highest proportion of di-nucleotide repeats (and highest and lowest proportion of tri-nucleotide repeats), respectively. The basis for the difference in frequency of repeat types across the sequenced tree species is not known, but is consistent with the range of variation observed across other plant species, when cross-species comparisons of EST-SSRs have been undertaken [44].

Respectively, 32 of the 44 and 22 of the 40 primer sets tested on the initial *F. albida* and *P. africana* test panels of 8 individuals revealed PCR products of the expected size (Table S2). For both species, nine of the primer sets tested resulted in larger than expected products, which may reflect the presence of intronic sequences in genomic amplifications. Of the primer sets that revealed products of the expected size and were therefore used to genotype 30 individuals of each species, ten (3 di- and 7 tri-nucleotide repeats) and eight (2 di- and 6 tri-nucleotide repeats) revealed easily-interpretable polymorphic products for *F. albida* and *P. africana*, respectively (information on product size range and number of alleles for these informative amplifications is given in Table S2). The average allele number per polymorphic locus was 3.4 in the case of *F. albida* and 4.5 in the case of *P. africana*.

## Discussion

Multiplexing based on bar-coding is an approach that is being increasingly applied during the next-generation sequencing of plants (see [25] for another example involving multiple tree species). Furthermore, EST-SSRs are the markers of choice for several population genetic applications and show greater transferability across taxonomic boundaries than SSRs derived simply from whole-genome DNA sequencing, which facilitates cross-species comparisons [37]. A comparison of the current NGS results (average of >5,000 EST-SSRs identified per species) with pre-existing National Center for Biotechnology Information of the USA (NCBI) citations indicates an enormous leap in resource availability through our study (Table 1). Only for two of our selected species, *A*. *mangium* and *J*. *curcas*, were significant prior sequence data available (>1,000 citations), explained in these cases by large-scale commercial interests in planting both species as well as them being of importance to smallholders. For the other 22 species, the average number of pre-existing NCBI nucleotide sequence citations was 57, with 20 species having fewer than 100 citations and 10 species fewer than 20 citations.

Information on sequences, EST-SSRs and putative primer pairs determined in the current study is presented in full at the tropiTree portal, where repeats and primer locations in transcripts are highlighted. From the portal, users can download sequence reads and SSR features for further examination within the Tablet template developed at JHI (available for download at: http://bioinf.hutton.ac.uk/tablet [12]). For a range of file formats, Tablet provides for whole-reference coverage overview, variant highlighting and paired-end read mark-up, among other features. Sorted BAM files for download from tropiTree range in size from 251 MB (*S. sesban* and *Z. mauritiana*) to 646 MB (*Enterolobium cyclocarpum*) (mean = 390 MB), while FASTA files range in size from 1.3 MB (*P. africana*) to 45 MB (*A. mangium*) (mean = 26 MB). The tropiTree portal also provides further methods for searching the results of sequencing, including by sequence homology via a BLAST search or by a keyword search of the TAIR annotations of the transcripts; these features should further enhance the use of data. As well as supporting research on the 24 tree species it currently contains, tropiTree provides a robust framework amenable for the addition, presentation and application of NGS data of further tropical trees.

Our examination of the utility of SSRs detected in the current study involved two species, *F. albida* and *P. africana*, for which prior population genetic studies [26]–[29] have not been able to draw on species-specific SSR markers. (In the case of *P*. *africana*, prior nSSR analysis by Kadu et al. 2013 [30] relied on markers derived from *Prunus avium* and *Prunus persica*, neither of which are native to Africa; BLASTN searches of global databases [Fig. S1, which also shows the results of BLASTX searches] revealed that the top hits for 50% of our *P. africana* transcripts were to the latter species; see also [43].) *Prunus africana* was chosen as one of the species for validation because it revealed by far the lowest number of transcripts and SSRs from sequencing, and it therefore provided the lower limit for the utility of our approach for marker development. Test screens indicated successful polymorphic marker recovery rates from putative primer pairs of 23% for *F. albida* and 20% for *P. africana.* These success rates are very similar to those recorded by Fu et al. [31], Liu et al. [32] and Wang et al. [39] for EST-SSRs derived from Illumina paired-end transcriptome sequencing of *Apium graveolens* (celery), *Medicago sativa* (alfalfa) and *Chrysanthemum nankingense* (chrysanthemum), respectively, suggesting similar levels of recovery can be expected from the sequences of the other tree species in our database. Success rates are however lower than those typically indicated by Schoebel et al. [11] for polymorphic SSR detection in 17 non-model species (plants, fungi, invertebrates, birds and a mammal) based instead on 454 pyrosequencing of genomic DNA.

With the very large number of putative primer pairs to SSRs available for testing in the tropiTree datasets – far more than required for most standard population genetic applications – we recommend that long repeats of motifs and high levels of repeat perfection are adopted as criteria in initial screening before primer testing [9]. Fernandez-Silva et al. [33] suggested other approaches for post-sequence pre-amplification microsatellite selection based on sequence quality and the avoidance of repetitive elements. Sequence annotation to detect SSRs in candidate genes of adaptive potential, or of other particular interest, is one useful approach that can be implemented both in tropiTree (e.g., see TAIR annotations given online, also illustrated in Table S2 [NB, a mean of 67% of SSR-containing transcripts had TAIR annotations, Table 1]) and in conjunction with tools such as Blast2GO [32], [34], [35], [38], [39]. Current tropiTree sequence data are a starting point for differential expression analysis (different tissues, conditions and time intervals) that may be most useful in classifying sequence functions (e.g., see [40]–[42] for recent tree examples).

## Final Remarks

tropiTree represents a significant and freely-available user-friendly resource for studies of gene flow, breeding systems, genetic diversity and population structure for a range of tropical trees important to rural communities, and provides a model for presenting tree NGS data to scientists. Sequencing technology is developing rapidly in terms of run output, read-length and lowered costs. Today (mid 2014), a single lane of HiSeq 2500 will generate up to 75 Gb of data and samples may now be indexed to a depth of 96 per lane, which would surpass the coverage per sample utilised in our current study (∼800 Mb compared to ∼500 Mb). Based on the typical current costs of service providers, this equates to only ∼£130 (∼220 USD) per species sample for sequencing. Thus, sequencing costs should now rarely, if ever, be a concern in marker development for non-model species. Rather, bioinformatic capacity and costs are now much more important, with tropiTree providing a useful model for presenting large data sets in a manner appropriate for population geneticists and others to use.

Finally, our data may also be used for single-nucleotide polymorphism (SNP) discovery in the sequenced species. Our experience, however, is that the detection of genuine SNPs based on data sets such as these of the current study is not straightforward and longer paired-end sequence reads would be preferable (see supplementary material to [36]). Screening of current sequences would require conservative application of read number and minimum minor allele frequency parameters, among other factors.

## Supporting Information

Figure S1 **BLASTN and BLASTX searches of** ***Prunus africana*** **transcripts.**
(TIF)

Table S1 **Information on source of RNA used for next-generation sequencing of 24 tropical tree species.**
(DOCX)

Table S2 ***Faidherbia albida*** **and** ***Prunus africana*** **SSRs for primer validation.**
(XLSX)
