# Supplementary figures and images for: tropiTree: An NGS-Based EST-SSR Resource for 24 Tropical Tree Species
Source: PLoS One. 2014 Jul 15;9(7):e102502. doi: 10.1371/journal.pone.0102502 (PMC4099372; doi:10.1371/journal.pone.0102502)

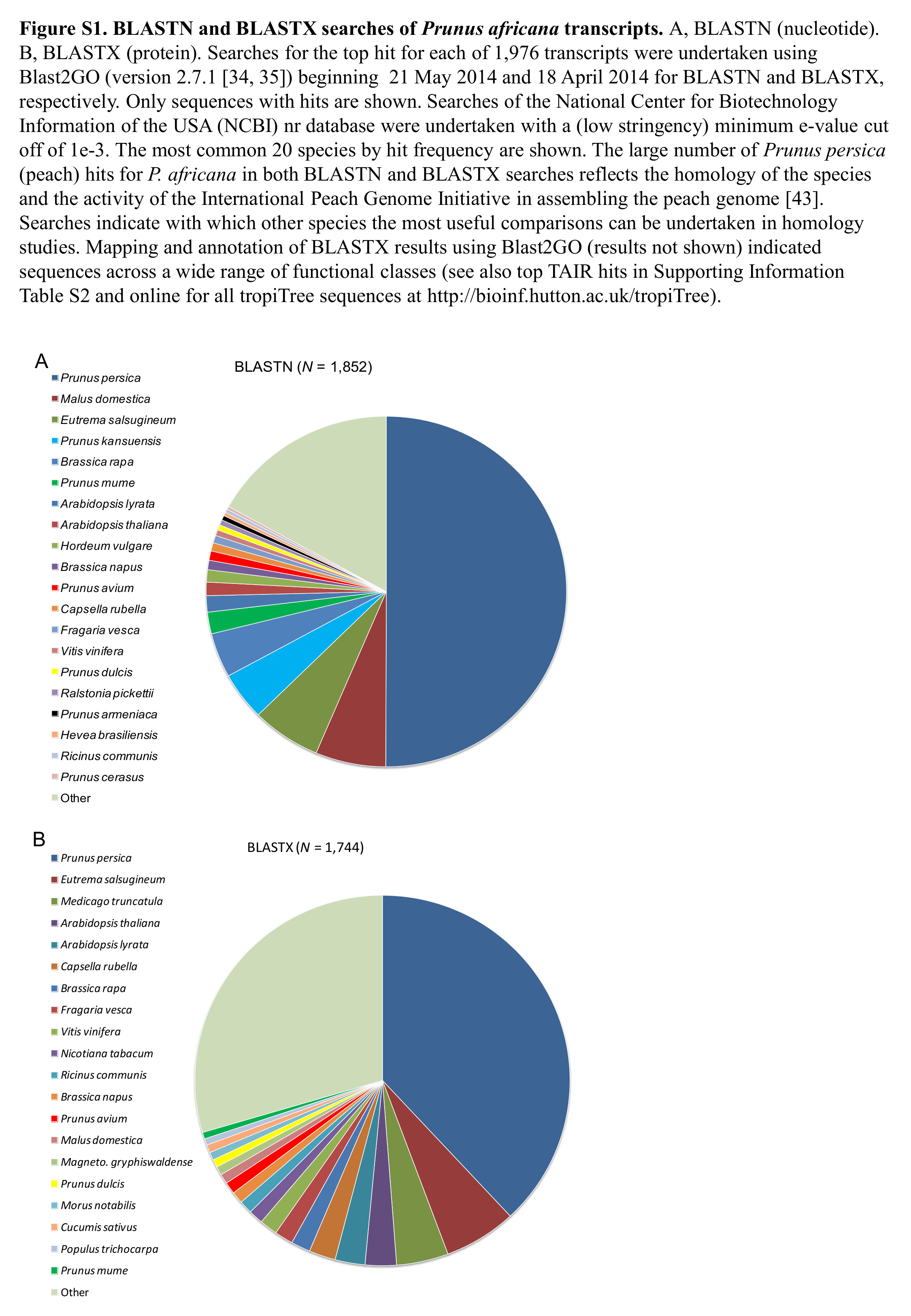

Supplement: Figure S1 — BLASTN and BLASTX searches of Prunus africana transcripts. (TIF) [file pone.0102502.s001.tif]
