# Supplementary material for: tropiTree: An NGS-Based EST-SSR Resource for 24 Tropical Tree Species
Source: PLoS One. 2014 Jul 15;9(7):e102502. doi: 10.1371/journal.pone.0102502 (PMC4099372; doi:10.1371/journal.pone.0102502)
Supplement: Table S1 — Information on source of RNA used for next-generation sequencing of 24 tropical tree species. (DOCX) [file pone.0102502.s002.docx]

**Supporting Information Table S1. Information on source of RNA used for next-generation sequencing of 24 tropical tree species.** Normally, embryonic tissue was pooled from 2 to 4 individuals to provide sufficient material for RNA extraction.

|  |  |
| --- | --- |
| **Species** | **Source of RNA** |
|  |  |
|  |  |
| *Acacia mangium* | Root only |
| *Acacia senegal* | Coleoptile only |
| *Acrocarpus fraxinifolius* | Root only |
| *Adansonia digitata* | Root only |
| *Albizia lebbeck* | Root only |
| *Calliandra calothyrsus* | Root only |
| *Diospyros mespiliformis* | Root only |
| *Enterolobium cyclocarpum* | Root only |
| *Faidherbia albida* | Root only |
| *Gliricidia sepium* | Root only |
| *Jacaranda mimosifolia* | Root only |
| *Jatropha curcas* | Root only |
| *Leucaena diversifolia* | Root only |
| *Leucaena leucocephala* | Root and embryo |
| *Moringa stenopetala* | Root only |
| *Prunus africana* | Root and coleoptile |
| *Samanea saman* | Root and embryo |
| *Senna siamea* | Root only |
| *Sesbania macrantha* | Root only |
| *Sesbania sesban* | Root only |
| *Tephrosia candida* | Root and coleoptile |
| *Tipuana tipu* | Root and coleoptile |
| *Warburgia ugandensis* | Root only |
| *Ziziphus mauritiana* | Root only |
|  |  |
